# Development of patient-reported outcomes item set to evaluate acute treatment toxicity to pelvic online magnetic resonance-guided radiotherapy

**DOI:** 10.1186/s41687-021-00326-w

**Published:** 2021-06-23

**Authors:** P. K. Møller, H. Pappot, U. Bernchou, T. Schytte, K. B. Dieperink

**Affiliations:** 1 Department of Oncology, Odense University Hospital, AgeCare, Academy of Geriatric Cancer Research, Odense University Hospital, Odense, Denmark; 2 Department of Clinical Research, University of Southern Denmark, Odense, Denmark; 3 Department of Oncology, Rigshospitalet, University Hospital of Copenhagen, Copenhagen, Denmark; 4 Department of Clinical Medicine, University of Copenhagen, Copenhagen, Denmark; 5 Laboratory of Radiation Physics, Odense University Hospital, Odense, Denmark; 6 Department of Oncology, Odense University Hospital, Odense, Denmark

**Keywords:** Patient-reported outcomes, PRO, Item selection, Cancer, Pelvic, Online MRgRT, Radiotherapy, MR-linac, Acute toxicity

## Abstract

**Background:**

A new technology in cancer treatment, the MR-linac, provides online magnetic resonance-guided radiotherapy (MRgRT) that combines real-time visualization of the tumor and surrounding tissue with radiation therapy to deliver treatment more accurately. Online MRgRT makes it possible to minimize treatment volume, potentially reducing acute treatment toxicity. Patient-reported outcomes (PRO) add the patient perspective to evaluating treatment toxicity related to new technology. The objective of this mixed-methods study was to develop and explore the content validity of a set of PRO items to evaluate acute pelvic toxicity to radiotherapy including online MRgRT.

**Methods:**

A literature review and chart audit were conducted to identify symptomatic adverse events (AEs) to be selected from the Patient-Reported Outcomes Version of the Common Terminology Criteria for Adverse Events (PRO-CTCAE) library and European Organisation for Research and Treatment of Cancer (EORTC) item library. To validate the content, the item set was applied in a prospective pilot cohort of patients referred for primary pelvic RT with curative intent. Patients reported symptoms weekly during RT (4–8 weeks) and the subsequent 4 weeks. Follow-up reports were collected at 8, 12, and 24 weeks after RT. To ensure symptom coverage clinician-reported toxicity and individual patient interviews were conducted. The symptomatic AEs were included in the final item set if ≥20% of patients reported them.

**Results:**

Eighteen acute symptomatic AEs were selected for the initial item set. Forty patients (32 prostate cancer, 8 cervical cancer) were included in the pilot study. Patients with prostate cancer and those with cervical cancer both reported all 18 acute AEs. However, vomiting was not reported by > 20% of patients thus excluded from the item set. Adding a few diagnosis-specific AEs to the final item set was required for both prostate and cervical cancer patients.

**Conclusions:**

A PRO item set for patients with pelvic cancer treated with radiotherapy with a curative intent was developed and content validity explored. In the pilot study, the item set captured the most common acute symptomatic AEs for patients with prostate and cervical cancer related to pelvic RT including online MRgRT. Further validation of the content in broader disease sites would be needed in future studies.

**Supplementary Information:**

The online version contains supplementary material available at 10.1186/s41687-021-00326-w.

## Background

Radiotherapy has advanced considerably during the past decades, improving survival and quality of life for cancer patients. Online magnetic resonance-guided radiotherapy (MRgRT), a recent innovation in radiation oncology, provides real-time visualization of the tumor and surrounding tissue during radiotherapy. It can increase disease control and survival with equivalent or decreased toxicity rates [1–3]. In 2018, the 1.5 T MR-linac (Unity, Elekta AB, Stockholm, Sweden) providing online MRgRT was ready for clinical use [4].

Until recently, external-beam radiotherapy for patients with pelvic cancer was guided by computed tomography (CT-guided) [3]. Online MRgRT is advantageous for these patients because the superior soft tissue differentiation of magnetic resonance imaging [5] in the pelvic area can reduce radiation exposure in healthy tissue [3, 6]. Treatment toxicity experienced by patients with pelvic cancer depends on the dose received and volume of irradiated healthy tissue [7–10].

Toxicity monitoring in cancer clinical trials is standardized prospective clinician reporting of the National Cancer Institute’s Common Terminology Criteria for Adverse Events (CTCAE), grading adverse events (AEs) on a scale from 0 to 5 [11]. CTCAE grading, as well as patient-reported outcomes [12], is part of the proposed standard assessment methodology for clinical evaluation of radiotherapy innovations like online MRgRT [13]. However, several studies have identified discrepancies between clinician and patient reporting in general oncology treatment. Clinicians appear to underreport the rate and severity of treatment toxicity, compared to patient-reported severity [14–18]. Patient self-reports are an important supplement to evaluating online MRgRT treatment tolerability, as in other oncological settings where they have been used as direct indicators of worsening, persistence or improvement of symptoms and general well-being [19–21]. Patient self-reports add a patient perspective to dose selection and may reduce the risk of undisclosed treatment toxicities.

Only four studies conducted in two sites have investigated patient-reported toxicity during and after online pelvic MRgRT [22–25]. All four studies used standardized validated questionnaires to measure acute PRO at predetermined time points: baseline, end of treatment and follow-up at week 6. Thus, assessment over time was based on few time points with a substantial gap from the end of treatment to 6 weeks after treatment completion, creating a risk of undetected increases in acute treatment toxicity. The authors recommended that future trials include earlier data collection points to map the trajectory of acute treatment toxicity [22]. They also called for a consensus on questionnaires used to capture radiotherapy treatment toxicity for prostate cancer patients because some relevant symptomatic AEs are missing in the standardized PRO questionnaires [23].

To capture the patient perspectives related to online MRgRT, it is important to ensure the right questions are asked. Questions reflecting relevant expected symptoms that are meaningful to the patients [26, 27]. A systematic selection of symptomatic AEs tailors the PRO questionnaire to the right purpose, population and treatment [28]. Selecting items addressing the identified symptomatic AEs is a way of choosing a minimum requirement of outcomes for a specific diagnosis and treatment [29]. Several core outcome set have been developed for pelvic cancer patients; however, the targeted treatment was not always specified nor were instruments for measuring core outcomes often addressed [29]. A previous study developed separate item sets for male and female pelvic radiotherapy patients targeted CT-guided radiotherapy based on interviews with a heterogeneous patient population including patients receiving palliative treatment [30]. Since online MRgRT allows us to enable dose escalation and reduce the treatment volume the incidence and severity of symptoms during the treatment trajectory may differ from standard treatment regimens [31–33]. As a consequence, a short, comprehensive item set is needed to capture weekly changes in the most common symptomatic AEs for patients throughout the treatment course. Symptoms that are not necessarily reported by clinicians, thus being valuable evidence in the evaluation of patient tolerance to online MRgRT. To our knowledge, no PRO item set is available to support the purpose of weekly monitoring of acute symptomatic AES to pelvic radiotherapy with a curative intent including online MRgRT. The objectives of this study were to: 1) identify symptomatic AEs for self-reporting for patients receiving primary pelvic radiotherapy with a curative intent and select equivalent items in validated item libraries and 2) evaluate the content validity of the prospective pilot study to ensure the item set covers the most common symptomatic AEs to pelvic radiotherapy including online MRgRT.

## Methods

### Study design

**Fig. 1:**
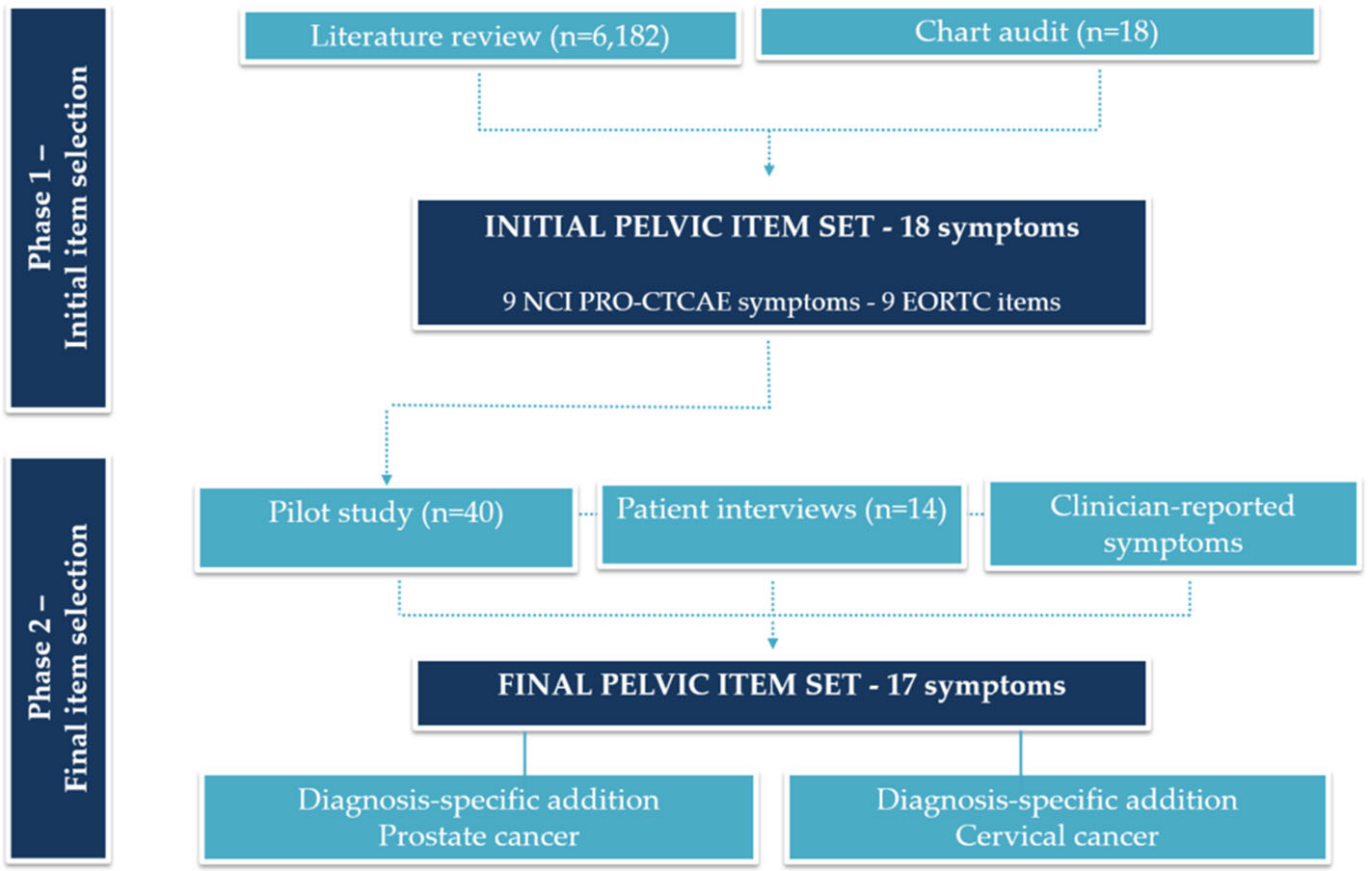
The item selection process

A mixed-methods approach included two phases: 1) initial item selection of relevant acute symptomatic AEs for primary pelvic radiotherapy and 2) a prospective pilot study applying the items selected in the first phase (Fig. 1). Methods used in the item selection process were inspired by systematic item selection as previously used [27, 30, 34–36]. A parallel mixed-methods approach was used to validate the content of the pelvic item set in phase 2 [37]. Data collection and analysis of qualitative and quantitative data occurred simultaneously, with findings synthesized in the final item selection.

### Phase 1: initial item selection

The initial item selection in phase 1 inspired by Tolstrup et al. [35] consisted of a literature review of acute toxicity to pelvic radiotherapy (rectal, cervical, urinary bladder and prostate cancer) and a chart audit of acute toxicity in patients treated with online MRgRT in the 1.5 T MR-linac at Odense University Hospital from the first patient in October 2018 until May 2019. The objective of the review and chart audit was to identify and map the most common acute symptomatic AEs among patients receiving primary pelvic radiotherapy from the start of radiotherapy until 6 months after completion.

A comprehensive literature search was carried out in June 2019 in the Cochrane, PubMed and Embase (Ovid) (Embase Classic+Embase 1947 to 2019 May 13), using Covidence to manage and sort references [38]. The search was guided by PRISMA guidelines [39] and an expert on literature searches reviewed the search terms. The literature search strategy is available in Additional file 1.

The purpose of the chart audit was to investigate acute symptoms reported by physicians, nurses and radiotherapists to supplement the literature review and assess the consistency of clinical reports with symptoms identified in review. Clinicians documented AEs in a pre-specified CTCAE form at fixed time points, and patient EHRs were searched to find additional symptoms reported at other times.

Acute symptomatic AEs found in the literature review and chart audit were included in the initial item set if they were reported: 1) in the literature for all four pelvic cancer diagnoses or 2) in the MRgRT EHR audit or the two clinical trials with online MRgRT and in the literature review for at least two diagnoses. After identifying initial prevalent symptomatic AEs, corresponding items were selected from validated item libraries. The Patient-Reported Outcomes version of Common Terminology Criteria of Adverse Events (PRO-CTCAE) developed by the National Cancer Institute (NCI) [40] and the European Organisation for Research and Treatment of Cancer (EORTC) item library provides a flexible collection of items [41]. The PRO-CTCAE item library comprises 124 items representing 78 symptomatic toxicities [40]. Some symptoms are not included in the PRO-CTCAE library, thus items were drawn from the EORTC item library to capture all relevant symptoms [41]. These two item libraries were used as they contain multiple items for patient self-reports of symptomatic AEs translated into Danish and tested for construct validity and reliability [41, 42]. When symptoms are available in both item libraries the wording of the item may influence the item selected.

### Phase 2: pilot study

The initial set of PRO items representing symptomatic AEs were applied in a prospective pilot study with patients treated at the Department of Oncology at Odense University Hospital in Denmark. The pilot study aimed to evaluate whether the pelvic item set addressed all relevant symptomatic AEs to pelvic radiotherapy including online MRgRT.

### Eligibility

All patients aged ≥18 years referred to the department for primary pelvic CT-guided RT or online MRgRT with a curative intent (rectal, cervical, urinary bladder or prostate cancer) in October 2019–June 2020 were eligible for inclusion. Patients were excluded if they were unable to give informed consent or to read, understand and respond to PRO questionnaires in Danish in electronic or paper formats. Sample size for the pilot study was set at 40 patients, based on sample sizes from previous pilot studies testing the integration of a PRO instrument into clinical cancer therapy [34, 43].

### Data collection period

Patients reported symptoms weekly during their four- to eight-week courses of radiotherapy and the subsequent 4 weeks. Seven days is the preferred recall period for the PRO-CTCAE items [44]. Follow-up reports were collected at 8, 12 and 24 weeks after completing radiotherapy. Data were collected from October 2019 to October 2020, at which time the study group completed the final item selection process. The patients were informed that their responses were not available for the clinicians in the pilot study.

### Variables

Demographic data on age and Eastern Cooperative Oncology Group/World Health Organization Performance Status (ECOG/WHO PS) [45] were obtained from the EHR, along with data on diagnoses, concomitant treatments, prostate risk group for patients with prostate cancer and the 2018 International Federation of Gynecology and Obstetrics (FIGO) staging system for patients with cervical cancer. In addition, data were collected on radiotherapy: dose absorbed in gray (Gy), number of radiotherapy fractions and whether online MRgRT was used.

#### Questionnaires and semi-structured interviews

The initial item set was supplemented by five questions for patient free text reporting of other symptoms experienced during treatment. The questions for free-text reporting of symptoms were available for patient-initiated reporting at any time.

The questionnaire was administered electronically through *My Hospital* or paper-based as an alternative. *My Hospital* is an app for patients at hospitals in the Region of Southern Denmark that enables patient-entered data to be shared with hospital clinicians through the EHR [46]. The app was used for data collection to support patient adherence to the reporting schedule because it was already in use in the oncology department to provide an overview of appointments and information about treatment. Patients received verbal and written instructions for reporting PRO in *My Hospital*. A paper-format questionnaire was offered to those not having a device or technical skills to report electronically.

The main investigator (PKM) obtained clinician-reported toxicity reported in the EHR by physicians and radiotherapists during radiotherapy and the subsequent 4 weeks. Individual interviews with patients were conducted using a convenience sampling method 1 month after treatment completion. The patients were interviewed in the chronological order they attended their 4-week follow-up continuing recruitment until no new information or themes emerged from the data and data saturation was reached [47]. A semi-structured interview guide was used to investigate whether the questions were clear and easy to respond to and whether all relevant symptoms they experienced were addressed by the questionnaire. To validate the content of the item set, patients were asked about any symptomatic AEs they experienced but not report in the electronic questionnaire. The symptomatic AEs were included in the final item set if ≥20% of patients reported them inspired by Sandler et al. [30].

### Statistical analyses

Sociodemographic and clinical characteristics of all patients were analyzed descriptively, as were the prevalence of items reported post-baseline and the proportion of other symptoms reported in scheduled and patient-initiated reports. All patients with pelvic cancer were included in the analysis. Study analyses were performed using STATA IC 15. Interview data were analyzed with systematic text condensation [48].

## Results

### Phase 1

**Fig. 2:**
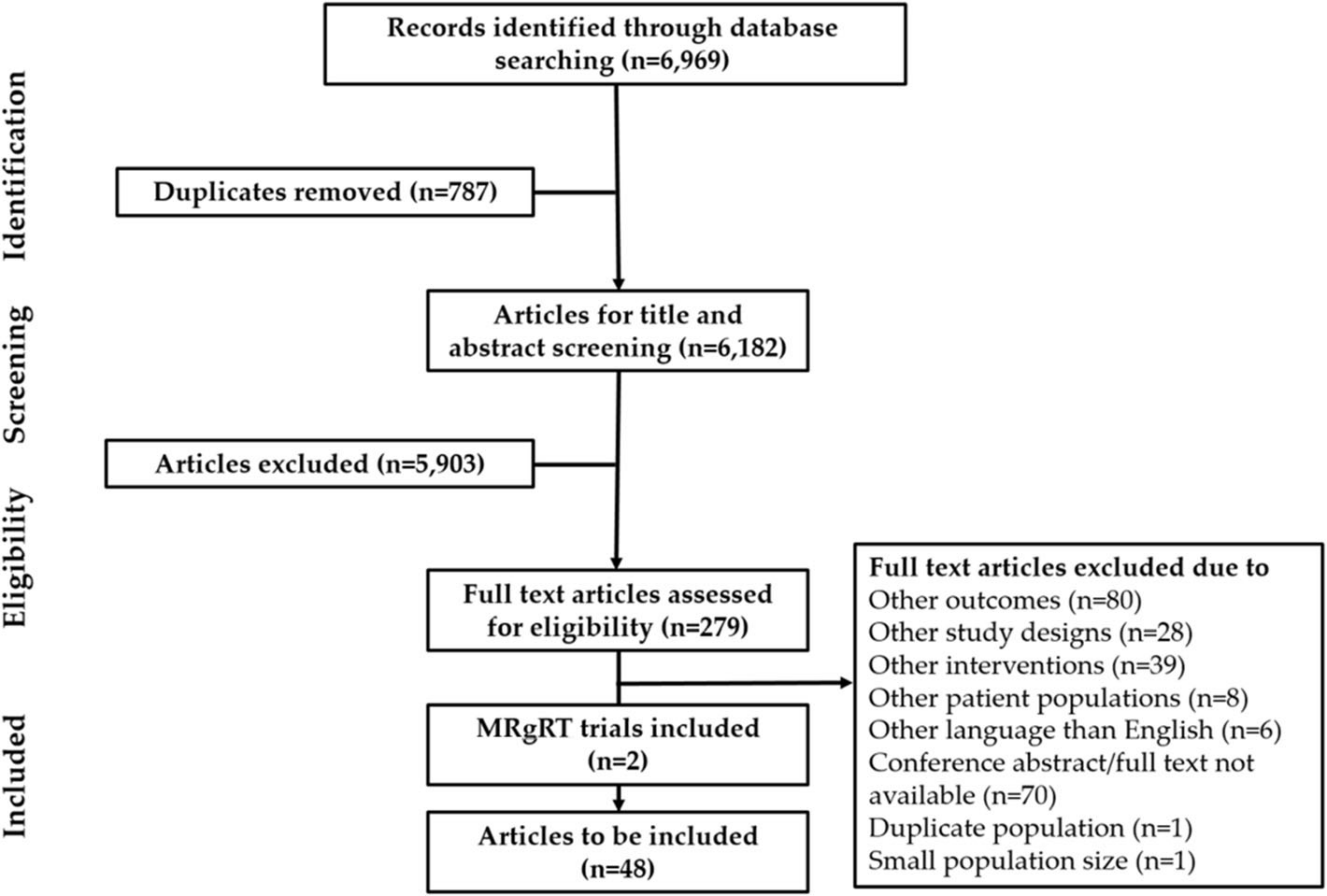
PRISMA diagram for literature review of acute toxicity to primary pelvic radiotherapy

In the initial item selection, 6182 articles were screened and 46 reports representing all four pelvic cancers were included in the final review (Fig. 2).

In addition, EHRs were reviewed for 18 patients with prostate cancer treated with online MRgRT (Fig. 1). No patients with other pelvic cancers were treated with online MRgRT. Two clinical trials published after the literature search on acute toxicity to online MRgRT in the pelvic region were added [22, 49] (Fig. 2). Thirty five acute symptomatic AEs that appeared in included literature, EHRs and trials were listed by the related CTCAE v. 5.0 term [50] (Additional file 2).

Ten symptomatic AEs reported by patients with each of the four types of pelvic cancer were selected as core symptoms for the item set. In addition, eight symptomatic AEs were reported by patients with at least two of the included cancer types and by patients receiving online MRgRT and/or in the two clinical MRgRT trials. Nine of the 10 core symptomatic AEs were available in the PRO-CTCAE item library; Fatigue, anorexia, radiation dermatitis, abdominal pain, constipation, diarrhea, nausea, urinary frequency and dysuria. Rectal pain was not in the PRO-CTCAE item library as well as three of the eight additional pelvic symptomatic AEs (urinary retention, nocturia, and rectal hemorrhage). A decision was made to select all supplemental items from the EORTC item library as these items covered the content of the identified symptoms better using a more plain language; Rectal pain, urinary retention, urinary incontinence, urinary urge, nocturia, vomiting, fecal incontinence, rectal hemorrhage and bloating. For some of the symptoms between one to three items were developed in the PRO-CTCAE library reflecting frequency, severity and interference. Consequently, the initial item set comprised 24 items from the PRO-CTCAE and EORTC item libraries addressing 18 symptomatic AEs.

### Phase 2

A total of 53 patients were eligible for inclusion in the pilot study after three patients were dismissed based on clinician assessment. Six patients were excluded due to starting RT during the lockdown of clinical trials because of COVID-19. Forty-seven patients were informed about the study. Six patients declined participation because they felt they lacked the resources to join a research study, and 41 patients agreed to participate. No patients with rectal cancer were referred to primary radiotherapy during the study period. One patient with bladder cancer was eligible and enrolled in the pilot study but excluded from the analysis due to this unique status. Forty patients were enrolled and included in the analysis: 32 with prostate cancer and eight with cervical cancer (Fig. 1). Thirty seven patients (93%) reported electronically. Median age was a little lower and with a wider range among the patients with cervical cancer compared to prostate cancer (Table 1). Compared to patients with prostate cancer, a smaller proportion of patients with cervical cancer scored zero (“fully active”) on ECOG/WHO performance status. Four patients with cervical cancer (50%) were also treated with weekly concomitant chemotherapy (Cisplatin) and 26 patients with prostate cancer (81%) were simultaneously treated with androgen deprivation therapy (ADT) (Table 1). In addition, 75% of the women (*n* = 6) had brachytherapy in their final week of external radiotherapy and again 1 or 2 weeks after radiotherapy completion (PDR-BT, 2 × 17.5 Gy/20 pulses). Of patients with prostate cancer, 13 (41%) were treated with online MRgRT. This treatment option was not yet available for patients with cervical cancer (Table 1).

**Table 1:** Characteristics of patients with pelvic cancer enrolled in the pilot study (*n* = 40)

Clinical data		All (*n* = 40)	Prostate (*n* = 32)	Cervix (*n* = 8)
Age, *median*	Years (range)	68 (36–76)	69 (54–76)	67 (36–75)
ECOG/WHO PS	0	33 (83%)	27 (84%)	6 (75%)
	1	6 (15%)	4 (13%)	2 (25%)
	2	1 (2%)	1 (3%)	0
Prostate risk group	Low risk		1 (3%)	*–*
	Intermediate risk		10 (31%)	*–*
	High Risk		21 (66%)	*–*
FIGO staging, cervical cancer	I		–	1 (13%)
	II		–	5 (62%)
	III		–	2 (25%)
Radiotherapy dose (Gy) /fractions	78/39	17 (42%)	17 (53%)	0
	62/21	1 (3%)	1 (3%)	0
	60/20	14 (35%)	14 (44%)	0
	55/25	2 (5%)	0	2 (25%)
	50/25	2 (5%)	0	2 (25%)
	46/26	1 (3%)	0	1 (12%)
	45/25	3 (7%)	0	3 (38%)
Online MRgRT	Yes	13 (33%)	13 (41%)	0
Brachytherapy (PDR-BT)	Yes		–	6 (75%)
Concomitant systemic treatment	Yes	30 (75%)	26 (81%)	4 (50%)

Compliance was high as 85% of the patients responded to > 80% of the weekly questionnaires. Reasons for non-compliance were the patients forgetting or not having the resources in that particular week due to fatigue or having many appointments in the clinic.

All 18 acute AEs were reported at some point during the weekly responses by patients with prostate cancer (Table 2) and those with cervical cancer (Table 3). Only one of the 18 symptoms, vomiting, had ≤20% prevalence among patients with prostate cancer (Table 2). No additional symptoms were reported by ≥20% of the patients or by clinicians for ≥20% of patients with either diagnosis. Therefore, only the symptomatic AE of vomiting was removed from the initial pelvic item set.

**Table 2:** Proportion of symptoms reported by patients with prostate cancer (n = 32)

Symptoms reported in weekly item set from baseline to follow-up week 4	Reported in pelvic item set, %	Reported in free-text or interview, %	Reported by clinicians in the patient chart, %
Nocturia	100	6	50
Urinary frequency	97		69
Fatigue	94	16	38
Diarrhea	94	3	38
Urinary retention	94	9	44
Urinary urgency	91	3	28
Painful urination	81	6	44
Bloating	78		13
Abdominal pain	75	6	16
Rectal pain	69	3	16
Faecal incontinence	66	3	6
Constipation	56		13
Decreased appetite	47	13	9
Urinary incontinence	47		13
Nausea	31	3	16
Radiation skin reaction	28	3	3
Blood in stools	28		13
Vomiting	13	3	3
*Other symptomatic AEs*			
Proctitis			19

**Table 3:** Proportion of symptoms reported by patients with cervical cancer (n = 8)

Symptoms reported in weekly item set from baseline to follow-up week 4	Reported in pelvic item set, %	Reported in free-text or interview, %	Reported by clinicians in the patient chart,%
Nocturia	88		
Urinary frequency	88		13
Fatigue	100	25	88
Diarrhea	100	25	63
Urinary retention	75		
Urinary urgency	88		
Painful urination	88	13	38
Bloating	100	13	
Abdominal pain	100	25	25
Rectal pain	100	13	25
Faecal incontinence	88		
Constipation	88		25
Decreased appetite	88		25
Urinary incontinence	50		
Nausea	100		88
Radiation skin reaction	88	38	0
Blood in stools	25	13	
Vomiting	88		38
*Other symptomatic AEs*			
Vaginal bleeding		38	13
Haemorrhoids		25	13
Vaginal pain		25	13

Diagnosis-specific additions were needed for both prostate and cervical cancer patients. Clinicians reported inflammation of the rectum (proctitis) for 19% of patients with prostate cancer at the end of treatment or 4 weeks later. Interviews revealed that patient-reported diarrhea arose from proctitis in some cases. Consequently, an item from the EORTC Proctitis module must be added when using the item set for patients with prostate cancer. For patients with cervical cancer, additional items were needed for symptoms of vaginal bleeding, vaginal pain and hemorrhoids and chemotherapy-related symptoms like vomiting if relevant. Abdominal pain was used to capture pain in the pelvic area. In addition, pain in the specific irradiated area was reported in free-text responses by 25% of patients with cervical cancer and 9% of patients with prostate cancer.

The 14 semi-structured interviews (11 patients with prostate cancer and three with cervical cancer) confirmed that the most relevant symptomatic AEs for their respective diagnosis were addressed by the pelvic item set. When directly asked about symptoms other than those included in the questionnaire, only a few additional symptoms were mentioned by < 20% of interview participants: memory loss and confusion, cystitis, weight gain/weight loss and symptoms related to systemic treatment.

## Discussion

To the best of our knowledge, this is the first study to define and test a PRO item set to assess weekly symptomatic AEs related to primary pelvic RT including online MRgRT. Literature review and patient charts were consistent in identifying the 18 most common acute symptomatic AEs. To capture all relevant symptomatic AEs, items were selected from two item libraries: PRO-CTCAE and EORTC item library. Previous studies have selected items from a single library [30, 34, 35], adding one or two items from other questionnaires. Our decision was based on the need to include all identified symptoms relevant to evaluating MRgRT treatment using items with a plain wording covering the content of the identified symptoms.

The international MR-linac Consortium has recommended using PRO-CTCAE for future prospective clinical trials to estimate treatment-induced toxicity; however, no specific items were suggested [13]. The pelvic item set follows the recommendation of being intended for the specific population investigated with the specific purpose of having a tool for prospective evaluation of acute treatment toxicity to online MRgRT [28].

The benefit of using an item set specifically developed for this purpose is that it captures the acute symptomatic AEs to RT with a 7 days recall period. In addition, using a simple item set for weekly PRO covering the most common symptomatic AEs rather than using several standardized questionnaires minimizes patient burden [51]. It ensures symptom coverage and relevance for this specific population in pelvic radiotherapy. Few proposals exist for measuring PRO when recommending symptomatic AEs in core outcome sets [29]. As a result, the pelvic item set reported here, tailored to the properties of online MRgRT, may enhance consistency in the measurement of identified acute symptomatic AEs. Tetar et al. [23] investigated PRO in online MRgRT and similarly pointed out that the standardized questionnaires they used were not developed for external radiotherapy and did not evaluate all relevant symptoms.

A single previous study defined disease site-specific item sets for PRO in pelvic radiotherapy [30]. Sandler et al. defined male and female pelvis item sets based on patient interviews. Among female patients, 10% had cervical cancer and 14% were in palliative treatment; among male patients, 30% had diagnoses other than prostate cancer [30]. We ended up including prostate and cervical cancer patients only, a rather homogenous group that was uniformly treated with curative intent. We experienced that the 17 most common symptomatic adverse events in the item set were similar for patients treated for prostate and cervical cancer. The additional symptomatic adverse events needed were related to the specific irradiated areas. Therefore, we find it relevant to have a generic pelvic item set supplemented by diagnosis-specific additions related to the irradiated area for the specific patient-group investigated rather than having gender-specific item set.

In Sandler et al. [30], item selection was based on interviews and a checklist of 40 items presented to patients during their last week of radiotherapy. Patients were asked to recall all symptoms they had experienced during radiotherapy. In contrast, we based the final item set on prospective weekly reports from baseline to 4 weeks after radiotherapy completion, limiting the risk of recall bias.

Clinicians reported proctitis for 19% of the patients with prostate cancer and interviews supported the need for a broader interpretation of proctitis without multiple proctitis symptoms being included in the item set. A review by Atkinson et al. investigating the association between CTCAE and PRO found poor agreement between well-validated PRO measures and clinician rating (CTCAE) for proctitis among patients with rectal or anal cancer [52]. A few years later, EORTC validated the first radiation proctitis-specific quality-of-life module (QLQ-PRT20) [53]. It comprises 21 items, some of which (i.e., rectal pain, diarrhea and rectal bleeding) were already included in the pelvic item set. To minimize response burden, we added one additional item (feeling unable to completely empty bowels) from this module. It is arguable whether these items accurately identify the prevalence of proctitis. However, if patients’ self-reports are used in communicating with clinicians during the course of radiotherapy, these symptomatic AEs may contribute to the assessment of proctitis [54].

Differences between female patients in the Sandler et al. study [30] and ours could potentially account for discrepancies in selected items. However, we agree with Sandler et al. that an additional PRO-CTCAE item covering pain in the irradiated area is relevant for patients undergoing radiotherapy as e.g. abdominal pain is not covering pain in different anatomical irradiated sites in the abdominal or pelvic area. The symptom nocturia was omitted by Sandler et al. [30] even though thirty-nine (98%) patients in our pilot study reported nocturia at some point during radiotherapy. However, this item is not in the PRO-CTCAE item library and would have needed to be selected from another item library or questionnaire, which may have led to its omission in the previous study. Another item omitted from the Sandler et al. female item set was urinary urgency, which was reported by 88% of cervical cancer patients in our pilot study. This illustrates why selecting PRO for a purpose is important [28].

As six women in the current study received brachytherapy parallel to external radiotherapy, this may have affected the severity of symptoms like vaginal pain among these patients in the weeks following radiotherapy completion. Since the purpose of this study was to validate if the item set captures the most common symptoms for patients with pelvic cancer during radiotherapy this does not affect the outcome, however, the need for diagnosis-specific additions for cervical cancer patients also receiving chemotherapy or brachytherapy should be investigated further in future studies.

Sexual health is relevant for patients with prostate cancer and Sandler et al. included three items related to sexual health in their male pelvic item set [30]. However, the site-specific item set was empirically established and validated for assessment of radiation-induced toxicity but not for weekly reporting during radiotherapy. The majority of patients with prostate cancer in the present pilot study had concomitant androgen deprivation therapy (ADT) for at least 6 months and also mentioned symptoms about sexual health in the weekly free-text responses, thus being constant throughout radiotherapy. In the following prospective study two PRO-CTCAE items covering sexual health will be added to baseline and follow-up PRO measures.

Study strengths included the systematic item selection process based on existing literature and the health records of the first patients receiving pelvic online MRgRT at our institution. Items were selected from item libraries to cover all relevant symptomatic AEs using items assessed for construct validity and reliability. Validating the content of the item set among members of the target population in a prospective pilot study is a major strength and is bolstered by the opportunity for patients and clinicians to add other symptoms. The multiple and regular scheduled assessment time points reflected the intended use of the PRO measures, providing optimal informational value [29].

Several limitations deserve mention. First, a limited number of patient charts were reviewed during initial item selection; however, they accounted for all the pelvic cancer patients being treated with online MRgRT at our institution at the time. To some extent, the use of mixed methods enhances the reliability of our findings. However, interviews were analyzed relatively superficially for the purpose of ensuring AE symptom coverage for the target patient population. An opportunity was missed to synthesize the quantitative findings with more detailed qualitative findings [37]. Further interview data analysis must be conducted to explore patient experience, acceptability and usability of integrating electronic PRO during radiotherapy. Finally, the content of the pelvic item set is validated only for patients with prostate cancer and a small sample of patients with cervical cancer. Only eight cervical cancer patients were enrolled, mainly due to Covid-19 enrollment restrictions and other competing research protocols. Inclusion of broader disease sites (bladder, vulvar, rectal and anal cancer) and higher sample sizes would be needed in future studies.

In future prospective clinical trials of online pelvic MRgRT, replacing standardized questionnaires with a rigorous pelvic PRO item set will support measuring the most relevant acute symptomatic AEs [23]. PRO-CTCAE free-text response options are available to capture unsolicited and unexpected symptoms that may occur due to differences in tumor size, radiotherapy dose or fractionation [40]. Using a systematic approach to item selection helps to ensure that the right questions are asked for the right purpose. Future trials must ensure that patient responses are acknowledged and used for individual symptom management in radiotherapy [55].

## Conclusion

A PRO item set for patients with pelvic cancer receiving radiotherapy with a curative intent was developed to capture expected and unanticipated symptoms of acute treatment toxicity related to online MRgRT in future prospective trials. Further validation of the content in broader disease sites would be needed in future studies. Diagnosis-specific items must be added to address all patient-reported symptoms.

## Supplementary Information

**Additional file 1.**

**Additional file 2.**

## Data Availability

The data supporting the findings presented in this article are available from Pia Krause Møller. However, data use was restricted to the current study and data are not publicly available. Literature search strings and chart audit notes are stored at the Department of Oncology at Odense University Hospital in Denmark and available from the corresponding author on reasonable request.

## Abbreviations

AE: Adverse event
CTCAE: Common Terminology Criteria of Adverse Events
PRO-CTCAE: Patient-Reported Outcomes Version of the Common Terminology Criteria for Adverse Events
PRO: Patient-Reported Outcome
ePRO: Electronic Patient-Reported Outcome
NCI: National Cancer Institute
EORTC: European Organization for Research and Treatment of Cancer
QLQ-C30: EORTC general core module
QoL: Quality of life
MR: Magnetic resonance
RT: Radiotherapy
MRgRT: Magnetic resonance guided radiotherapy
Gy: Gray
ECOG: Eastern Cooperative Oncology Group
WHO: World Health Organization Performance Status
FIGO: 2018 International Federation of Gynecology and Obstetrics staging
