# Correction to: Development of patient-reported outcomes item set to evaluate acute treatment toxicity to pelvic online magnetic resonance-guided radiotherapy

**DOI:** 10.1186/s41687-021-00333-x

**Published:** 2021-07-27

**Authors:** P. K. Møller, H. Pappot, U. Bernchou, T. Schytte, K. B. Dieperink

**Affiliations:** 1 Department of Oncology, Odense University Hospital, AgeCare, Academy of Geriatric Cancer Research, Odense University Hospital, Odense, Denmark; 2 Department of Clinical Research, University of Southern Denmark, Odense, Denmark; 3 Department of Oncology, Rigshospitalet, University Hospital of Copenhagen, Copenhagen, Denmark; 4 Department of Clinical Medicine, University of Copenhagen, Copenhagen, Denmark; 5 Laboratory of Radiation Physics, Odense University Hospital, Odense, Denmark; 6 Department of Oncology, Odense University Hospital, Odense, Denmark

**Correction to: Journal of Patient-Reported Outcomes 5, 47 (2021)**

**https://doi.org/10.1186/s41687-021-00326-w**

Following publication of the original article [1], the authors identified an error in the authors and affiliations lists.

The current authors and affiliations lists read:

P. K. Møller1,2, H. Pappot3,4, U. Bernchou2,5, T. Schytte2,6, K. B. Dieperink1,2 and Pia Krause Møller7*

Department of Oncology, Odense University Hospital, AgeCare, Academy of Geriatric Cancer Research, Odense University Hospital, Odense, Denmark. 2 Department of Clinical Research, University of Southern Denmark, Odense, Denmark. 3 Department of Oncology, Rigshospitalet, University Hospital of Copenhagen, Copenhagen, Denmark. 4 Department of Clinical Medicine, University of Copenhagen, Copenhagen, Denmark. 5 Laboratory of Radiation Physics, Odense University Hospital, Odense, Denmark. 6 Department of Oncology, Odense University Hospital, Odense, Denmark. 7 Odense University Hospital, Research Unit of Oncology, Kløvervænget 19, 5000 Odense C, Denmark

The correct authors and affiliations lists should read

P. K. Møller1,2, H. Pappot3,4, U. Bernchou2,5, T. Schytte2,6, K. B. Dieperink1,2

Department of Oncology, Odense University Hospital, AgeCare, Academy of Geriatric Cancer Research, Odense University Hospital, Odense, Denmark. 2 Department of Clinical Research, University of Southern Denmark, Odense, Denmark. 3 Department of Oncology, Rigshospitalet, University Hospital of Copenhagen, Copenhagen, Denmark. 4 Department of Clinical Medicine, University of Copenhagen, Copenhagen, Denmark. 5 Laboratory of Radiation Physics, Odense University Hospital, Odense, Denmark. 6 Department of Oncology, Odense University Hospital, Odense, Denmark.

The author group has been updated above and the original article [1] has been corrected.
