# Supplementary material for: Development of patient-reported outcomes item set to evaluate acute treatment toxicity to pelvic online magnetic resonance-guided radiotherapy
Source: J Patient Rep Outcomes. 2021 Jun 23;5:47. doi: 10.1186/s41687-021-00326-w (PMC8220120; doi:10.1186/s41687-021-00326-w)
Supplement: Supplementary file 1 — Additional file 1. [file 41687_2021_326_MOESM1_ESM.docx]

**Literature Search Strategy**

#### Eligibility criteria

The eligibility criteria for including and excluding studies in the review were pre-specified. The literature was found eligible if it fulfilled the following criteria:

1. Patients with cancer rectum, uterine cervical cancer, urinary bladder cancer or prostate cancer as their primary treatment
2. Treated with conventional external radiotherapy in linear accelerators
3. Treated with a curative intent (not palliative treatment)
4. Literature with acute toxicity during/after radiotherapy (<= 6 months). Excluded if acute toxicity not specified.
5. Research from the past 5 years (2014-2019)
6. Literature in English
7. Excluded if case study, teaching courses, conference papers, protocols
8. Excluded if only abstract available

**Information sources**

Cochrane, PubMed, Embase (Ovid) (Embase Classic+Embase 1947 to 2019 May 13).

Date of literature search: 12.06.2019

**Cochrane**

| Radiotherapy | Adverse effects | Patients with pelvic cancer |
| --- | --- | --- |
| Radiotherapy  OR  radiotherap*  OR  *radiat**  *OR*  *radiation therapy*  *OR*  *radiation treatment*  *OR*  *irradiat** | Radiation injuries  *OR*  *radiation effect**  *OR*  *side-effect**  *OR*  *adverse effect**  *OR*  *adverse event**  OR  *symptom** | Pelvic Neoplasms  OR (Pelvic AND (cancer OR tumor OR neoplasm* OR tumor* OR tumour*))  Prostatic Neoplasms OR  (Prostae* AND (cancer OR tumor OR neoplasm* OR tumor* OR tumour*))  Uterine Cervical Neoplasms(MeSH)  ((Cervi* OR uteri* OR gynaecolog*) AND (cancer OR tumor OR neoplasm* OR tumor* OR tumour*))  Rectal neoplasms(MeSH)  (Rect* AND (cancer OR tumor OR neoplasm* OR tumor* OR tumour*))  Urinary Bladder Neoplasms(MeSH)  (Bladder* AND (cancer OR tumor OR neoplasm* OR tumor* OR tumour*)) |

**Pubmed**

| Radiotherapy | Adverse effects | Patients with pelvic cancers |
| --- | --- | --- |
| Radiotherapy  OR  radiotherap*  OR  radiat*  OR  radiation therap*  OR  radiation treatment*  OR  irradiat* | Radiation Injuries  OR  radiation effects  OR  signs and symptoms  OR  Adverse effects  OR  *Side-effect**  *OR*  *adverse effect**  *OR*  *adverse event**  *OR*  *symptom** | Pelvic Neoplasms  OR (Pelvic AND (cancer OR tumor OR neoplasm* OR tumor* OR tumour*))  Prostatic Neoplasms OR  (Prostae* AND (cancer OR tumor OR neoplasm* OR tumor* OR tumour*))  Uterine Cervical Neoplasms(MeSH)  ((Cervi* OR uteri* OR gynaecolog*) AND (cancer OR tumor OR neoplasm* OR tumor* OR tumour*))  Rectal neoplasms(MeSH)  (Rect* AND (cancer OR tumor OR neoplasm* OR tumor* OR tumour*))  Urinary Bladder Neoplasms(MeSH)  (Bladder* AND (cancer OR tumor OR neoplasm* OR tumor* OR tumour*)) |

**Embase** (Ovid)

| Radiotherapy | Adverse effects | Patients with pelvic cancer |
| --- | --- | --- |
| hypofractionated radiotherapy/ or external beam radiotherapy/ or (radiotherap* or radiation or irradiat*).mp. or conformal radiotherapy/ or exp radiotherapy/ | Radiation response.mp. or radiation response/ or radiation injury.mp. or radiation injury/ or adverse effect.mp. or adverse event/ or acute toxicity/rt [Radiotherapy] or side-effect.mp. | pelvic tumor.mp. or pelvis tumor/rt [Radiotherapy]  prostate cancer.mp. or prostate cancer/rt [Radiotherapy]  uterine cervical cancer.mp. or uterine cervix cancer/rt [Radiotherapy]  rectal neoplasm.mp. or rectum tumor/rt [Radiotherapy]  urinary bladder cancer.mp. or bladder cancer/rt [Radiotherapy] |
