# Supplementary material for: Development of patient-reported outcomes item set to evaluate acute treatment toxicity to pelvic online magnetic resonance-guided radiotherapy
Source: J Patient Rep Outcomes. 2021 Jun 23;5:47. doi: 10.1186/s41687-021-00326-w (PMC8220120; doi:10.1186/s41687-021-00326-w)
Supplement: Supplementary file 2 — Additional file 2. [file 41687_2021_326_MOESM2_ESM.docx]

| **Acute symptomatic adverse events from the literature search and patient charts** | |
| --- | --- |
| **General disorders and administration sites conditions** | Fatigue |
|  | Pain |
| **Gastrointestinal disorders** | Abdominal pain |
|  | Bloating |
|  | Constipation |
|  | Diarrhea |
|  | Faecal incontinence (urge) |
|  | Flatulence |
|  | Nausea |
|  | Proctitis |
|  | Rectal hemorrhage |
|  | Rectal pain |
|  | Vomiting |
|  | Hemorrhoid |
|  | Rectal obstruction |
| **Metabolism and nutrition disorders** | Anorexia (weight loss) |
| **Renal and urinary disorders** | Bladder spasm |
|  | Hematuria |
|  | Urinary frequency |
|  | Urinary incontinence |
|  | Urinary retention |
|  | Urinary urgency |
|  | Straining |
|  | Nocturia |
|  | Painful urination (dysuria) |
| **Vascular disorders** | Radiation skin reaction |
| **Reproductive system and breast disorders** | Erectile dysfunction |
|  | Ejaculation disorder |
|  | Anorgasmia |
|  | Vaginal dryness |
| **Psychiatric disorders** | Depression |
|  | Insomnia |
|  | Libido decreased |
|  | Hot flashes |
